# ‘Treatment resistance’ in electroconvulsive therapy (ECT) patients: time to move on

**DOI:** 10.1111/acps.13091

**Published:** 2019-10-25

**Authors:** C. H. Kellner, A. Nordenskjöld

**Affiliations:** ^1^ Electroconvulsive Therapy (ECT) New York Community Hospital Brooklyn NY USA; ^2^ Department of Psychiatry Icahn School of Medicine at Mount Sinai New York NY USA; ^3^ Faculty of Medicine and Health University Health Care Research Center Örebro University Örebro Sweden

The concept of ‘treatment resistance’ has become all the rage in depression research. It is used to define populations in research studies and in treatment algorithms as a rationale for moving on from standard antidepressants to other therapies. In such algorithms, electroconvulsive therapy (ECT) is often bundled improperly with less effective neurostimulation‐methods and experimental pharmacotherapies. We question the utility of the concept of ‘treatment resistance’ for research and for treatment selection for individual patients; we also have concerns about how it is interpreted by patients and their families. In a recent paper, one of us (AN) and colleagues opted to describe the patient population in the study as having had ‘less (or more) pharmacological treatment’, rather than always characterizing them as less, or more, ‘treatment resistant ‘^1^. We believe this is a more accurate characterization because it sticks to describing the facts and does not make any etiological assumptions.

The concept behind the term ‘treatment resistant’ is that certain patients have an intrinsically more difficult illness to treat, either because it is more severe or because it has biological characteristics that render standard treatments less effective 2. Does the number of treatment trials that a patient has been given really prove that they have such an illness? There are many variables determining the interactions between patients and the healthcare system, clearly not all related to intrinsic illness properties. In research, exposure to pharmacotherapies is relevant but insufficient to describe the severity or complexity of a patient population, and the problematic term ‘treatment resistance’ only adds confusion.

In our clinical work, patients who are referred for ECT usually have been exposed to multiple antidepressant trials and are thus considered ‘treatment resistant’, yet a majority still respond to ECT. Nevertheless, ‘treatment resistance’ should neither be sufficient nor necessary to refer a patient for ECT. Severity and profile of symptoms, episodicity, comorbidity, age, and family history of psychiatric illness are just a few factors that are more important to consider when identifying the biological illness for which ECT is the best treatment 3. It is not ethical to extend the suffering and to jeopardize the safety of a patient with psychotic or suicidal depression by insisting on several ‘adequate’ pharmacotherapy trials with uncertain outcome, merely to fulfill a treatment algorithm's requirement for referral. Furthermore, most ECT patients would not have been labeled ‘treatment resistant’ in the first place had they been offered ECT as a first‐ or second‐line treatment option. We believe that simply stating the facts of the patient's treatment history is sufficient and more accurate than labeling them ‘treatment resistant’. We like the terms ‘prior treatment exposure’ and ‘prior treatment history’.

There is, of course, yet another theoretical possibility: that multiple drug exposures do, in fact, change the biology of the patient's illness, causing their symptoms to be harder to treat, destabilizing the illness. Again, such a patient might have responded to ECT early on in the course of their illness and never have become ‘treatment resistant’.

There is also the possibility that a patient is ‘treatment resistant’ because he or she has a chronic complex psychiatric illness. In ECT practice, we often see patients who are referred because practitioners are desperate and ‘out of options’ for another medication treatment trial. They, and the patient, are led to believe that ECT is their ‘last resort’. Such patients typically have serious, complicated psychiatric histories, with multiple comorbidities. Their primary illness is not a mood or psychotic disorder, but is rather more driven by personality, psychosocial and environmental issues; for them, ECT may not be an appropriate option 4, 5. They need intensive, ongoing multimodal psychiatric care, typically with medication combinations and psychotherapy, but not usually ECT.

Finally, in the dialogue between physicians, patients, and significant others, we need to be careful with labels. We should use words that promote hope and de‐stigmatize the illness. We can only guess how a depressed patient interprets the doctor's verdict that he or she is ‘treatment resistant’. If it discourages the patient from trying another treatment, it may be deleterious.

We believe that, for the ECT patient population at least, the field would be better served to move away from the fraught concept and term ‘treatment resistant’. Better to just describe what prior treatment trials have been prescribed in the current episode, and leave it at that.

## Declaration of interest

Dr. Kellner reports personal fees from UpToDate, Psychiatric Times, Northwell Health and Cambridge University Press. Dr. Nordenskjöld reports no conflicts of interest.
